# Interlimb Generalization of Learned Bayesian Visuomotor Prior Occurs in Extrinsic Coordinates

**DOI:** 10.1523/ENEURO.0183-18.2018

**Published:** 2018-08-08

**Authors:** Christopher L. Hewitson, Paul F. Sowman, David M. Kaplan

**Affiliations:** 1 Department of Cognitive Science; 2 ARC Centre of Excellence in Cognition and its Disorders; 3 Perception in Action Research Centre, Macquarie University, Sydney, 2109, Australia

**Keywords:** Bayesian integration, interlimb generalization, motor learning, sensorimotor learning, transfer, visuomotor adaptation

## Abstract

Recent work suggests that the brain represents probability distributions and performs Bayesian integration during sensorimotor learning. However, our understanding of the neural representation of this learning remains limited. To begin to address this, we performed two experiments. In the first experiment, we replicated the key behavioral findings of Körding and Wolpert (2004), demonstrating that humans can perform in a Bayes-optimal manner by combining information about their own sensory uncertainty and a statistical distribution of lateral shifts encountered in a visuomotor adaptation task. In the second experiment, we extended these findings by testing whether visuomotor learning occurring during the same task generalizes from one limb to the other, and relatedly, whether this learning is represented in an extrinsic or intrinsic reference frame. We found that the learned mean of the distribution of visuomotor shifts generalizes to the opposite limb only when the perturbation is congruent in extrinsic coordinates, indicating that the underlying representation of learning acquired during training is available to the untrained limb and is coded in an extrinsic reference frame.

## Visual Abstract

**Figure:**
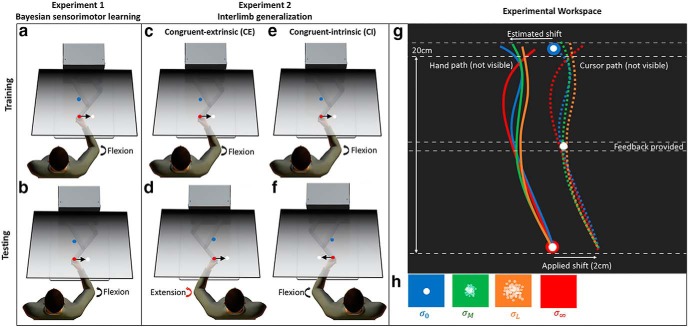

## Significance Statement

Generalization provides unique insights into the motor learning process. However, this type of learning has typically been investigated using fixed or deterministic perturbations and noise-free feedback information, which are not naturalistic. Here, we replicate important findings indicating that information is integrated in a Bayes-optimal manner during sensorimotor learning under uncertainty. We then extend these findings by showing that this learning generalizes to the opposite limb. These results have implications for our understanding of the neural mechanisms of motor learning as well as practical application to the contexts of sport training and motor rehabilitation.

## Introduction

Mounting neural, behavioral, and computational evidence suggests that the brain encodes probability distributions and performs probabilistic or Bayesian inference (Rao et al., 2002; Knill and Pouget, 2004; Rao, 2004; Ma et al., 2006; Doya, 2007; Pouget et al., 2013). The Bayesian coding hypothesis (Knill and Pouget, 2004) has been tested primarily in the context of perception (Knill and Richards, 1996; Rao et al., 2002; Weiss et al., 2002; Kersten and Yuille, 2003; Adams et al., 2004; Stocker and Simoncelli, 2006) and multisensory integration (van Beers et al., 1996, 1999; Alais and Burr, 2004; Ernst and Banks, 2002; Ma et al., 2006, Ma and Pouget, 2008; Beierholm et al., 2009; Fetsch et al., 2009, 2011, 2013). However, it has also been investigated in studies of sensorimotor learning, albeit to a lesser extent (Körding and Wolpert, 2004; Tassinari et al., 2006; Izawa and Shadmehr, 2008; Fernandes et al., 2012, 2014). In a seminal study, Körding and Wolpert (2004) demonstrate that subjects can learn to adapt their reaches to the mean of a probability distribution of visual shifts encountered in a modified visuomotor adaptation paradigm and, importantly, can regulate their dependence on this learned distribution according to the current level of sensory uncertainty in the feedback they are provided. This pattern of results is consistent with subjects performing Bayesian estimation during sensorimotor learning. Surprisingly, no subsequent studies have replicated this important finding and only a few have sought to test related questions or extend the paradigm (Wei and Körding, 2010; Fernandes et al., 2012, 2014). In this article, we report on an approximate replication of the original Körding and Wolpert (2004) study and an extension to the context of interlimb generalization (IG).

Generalization, which refers to the process by which experience or training in one context changes performance in another, provides a useful window into the representational changes underlying various forms of sensorimotor learning (Thoroughman and Shadmehr, 2000; Poggio and Bizzi, 2004; Shadmehr, 2004; Paz and Vaadia, 2009). Sensorimotor learning been shown to generalize across similar tasks or conditions using the same limb (intralimb generalization) and across limbs (IG). The extent to which learning generalizes is typically thought to reflect a common neural representation. By evaluating error patterns during generalization, inferences can be made about the reference frame for movement planning and control (Krakauer et al., 1999; Shadmehr, 2004).

With respect to intralimb generalization, it has been repeatedly shown that subjects who learn to adapt their movements in response to altered visual feedback for a restricted set of movement directions can generalize this learning to untrained directions (Bedford, 1989; Flanagan and Rao, 1995; Ghilardi et al., 1995; Wolpert et al., 1995; Ghahramani et al., 1996; Krakauer et al., 1999, 2000; Vetter et al., 1999; Malfait et al., 2002; Wu and Smith, 2013). A general finding is that visuomotor learning is represented in extrinsic (e.g., screen-based) coordinates (Cunningham and Welch, 1994; Imamizu and Shimojo, 1995; Krakauer et al., 1999, 2000; Vetter et al., 1999; Ghez and Krakauer, 2000). IG studies similarly indicate that visuomotor perturbations are represented and learned in extrinsic coordinates (Wang and Sainburg, 2003, 2004). However, several recent studies indicate that a combination or mixture of reference frames may be involved (Sober and Sabes, 2003, 2005; McGuire and Sabes, 2009; Brayanov et al., 2012; Carroll et al., 2014; Poh et al., 2016).

Interestingly, Körding and Wolpert (2004) tested neither form of generalization in their original study, but instead only probed whether subjects could learn the initial training task. Relatively little is known about how prior learning of a stochastic visuomotor perturbation involving a probability distribution of visuomotor rotations or shifts generalizes (Tassinari et al., 2006; Fernandes et al., 2012, 2014). In one of the most pertinent studies to date, Fernandes et al. (2012) investigated how different learned visuomotor priors generalize to new reach directions by having separate groups of subjects adapt to different distributions of visuomotor rotations with the same mean (30°) but different standard deviations (SD = 0°, 4°, or 12°). They found that learning was slower and less complete when the SD of the imposed distribution was higher, but interestingly the generalization curves were unaffected. In a subsequent study, Fernandes et al. (2014) replicated their earlier intralimb generalization findings and also showed that reliance on visual feedback about the current perturbation (the likelihood distribution) is greater when the prior distribution of visuomotor rotations imposed during training is wider and therefore associated with more uncertainty. Despite these investigations, generalization of a statistical prior across limbs has not been tested.

In the current study, we test the hypothesis that the mean of a distribution of stochastic visuomotor perturbations learned with one limb generalizes to the other limb. Based on analogous studies involving deterministic visuomotor perturbations, we predict that the representation of this learning is encoded in extrinsic coordinates.

## Materials and Methods

### Participants

A total of 35 right-handed subjects (22 males, 13 females, age 17–49 years) with normal or corrected to normal vision and no history of motor impairments participated in the experimental study. All subjects gave informed consent before the experiment and were either paid and recruited from the University’s Cognitive Science Participant Register or were University students participating for course credit. All experimental protocols were approved by the University’s Human Research Ethics Committee (protocol number: 5201600282). Subjects were randomly assigned to one of three experimental groups. Seven subjects participated in experiment 1, which consisted of a pseudo-replication of Körding and Wolpert (2004)’s stochastic visuomotor adaptation task. Fourteen subjects participated in experiment 2, which sought to test IG of visuomotor learning using the same basic task from experiment 1, but with an additional set of task conditions designed to test the extent to which learning with one limb is available to the untrained limb and the nature of the reference frame in which the initial learning occurs. Seven subjects also participated in an additional control for experiment 1, which consisted of a variation of the basic visuomotor adaptation task used in experiment 1.

### Experimental procedures

**Figure 1.**
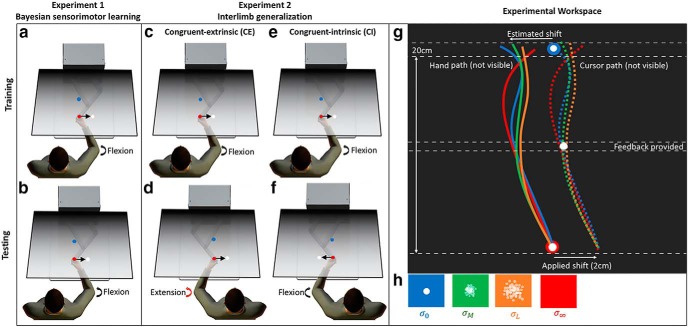
***A–F***, Experimental paradigm. ***G***, Experimental workspace with example hand and cursor paths shown for a representative trial when a 2-cm lateral visual shift is applied. Dashed white lines indicate feedback windows. ***H***, Midpoint feedback conditions with different amounts of visual uncertainty. Panels ***G***, ***H*** after Körding and Wolpert (2004).

A unimanual KINARM endpoint robot (BKIN Technologies) was used in all experiments (Fig. 1). The KINARM robot has a single graspable manipulandum that permits unrestricted 2D arm movement in a horizontal 2D plane (the movement plane). A projection-mirror system facilitates presentation of visual stimuli that appear in the movement plane. Subjects received visual feedback about their hand position via a cursor (solid white circle, 1 cm in diameter) that was controlled in real time by moving the manipulandum. Mirror placement and an opaque apron attached to the participant ensured that visual feedback from the real hand was not available for the duration of the experiment.

Although Körding and Wolpert (2004) had subjects perform optically-tracked pointing movements on a horizontal table surface rather than reaches with a robotic manipulandum, the task kinematics in the current study were closely matched. The task dynamics were also similar since the small changes produced by the manipulandum with respect to inertia and friction are negligible compared to that of the arm itself during unrestricted reaching and pointing movements. More generally, robotic manipulanda have been successfully used to investigate visuomotor adaptation in a number of previous studies (Saijo and Gomi, 2010; Hayashi et al., 2016; Leow et al., 2017) including studies highly similar to the current one (Wei and Körding 2010).

Subjects were instructed to perform fast and accurate goal-directed reaching movements with the dominant (right) arm using cursor feedback whenever it was available. Reaches were from a start target (solid red circle, 1 cm in diameter) located at the center of the workspace to a single reach target (solid blue circle, 1 cm in diameter) located 20 cm away (Fig. 1*G*). When subjects moved the cursor within the boundaries of the start target its color changed from blue to red and the reach target appeared, indicating the start of a trial. Subjects were free to reach at any time after the start target color changed. Once the cursor exited the start target, cursor feedback was extinguished and laterally shifted to the right of the true hand position (positive in the *x*-plane) by an amount drawn at random on each trial from a Gaussian distribution with mean of 1 cm and SD of 0.5 cm (the true prior). At the midpoint of the movement, displaced cursor feedback was provided for 100 ms (midpoint feedback).

To test whether Bayesian integration occurs during sensorimotor learning, following Körding and Wolpert (2004), the reliability of the sensory feedback information provided about the true cursor position at the reach midpoint was varied by introducing different amounts of visual noise or blur on each trial. Changing the degree of uncertainty associated with the current sensory evidence (the likelihood) allowed us to assess the subjects’ reliance on their previously experienced distribution of shifts (the prior). One of four visual uncertainty conditions ($\sigma_{0}$, $\sigma_{M}$, $\sigma_{L}$, $\sigma_{\infty }$; Fig. 1*H*) was selected at random on each trial according to a ratio of 3:1:1:1 previously used by Körding and Wolpert (2004). In the zero uncertainty condition ($\sigma_{0}$), midpoint feedback was a single white sphere (1 cm in diameter), identical to the initial cursor. In the moderate uncertainty condition ($\sigma_{M}$), midpoint feedback was one of ten randomly generated point clouds comprised of 50 small translucent white spheres (0.2 cm in diameter) distributed as a two-dimensional Gaussian with a SD of 1 cm and a mean centered over the true (displaced) cursor position on the current trial. In the large uncertainty condition ($\sigma_{L}$), everything was the same as the moderate uncertainty condition ($\sigma_{M}$) except that the point clouds had a SD of 2 cm. In the unlimited uncertainty condition ($\sigma_{\infty }$), no midpoint feedback was provided. Cursor feedback was again extinguished for the remainder of the reach to the end target. Cursor feedback at the endpoint of the reach (endpoint feedback) was provided only in the zero uncertainty ($\sigma_{0}$) condition for a duration of 100 ms. After movement offset, there was a delay of 150 ms before the start target reinitialized the next trial by changing color from red back to blue. The maximum allowable time to complete a reach was 4000 ms. Irrespective of the cursor’s position along the *x*-axis, if subjects did not cross the lower bound of the end target along the *y*-axis (Fig. 1*G*, dashed line) the trial would time out. Timeouts were signaled by the disappearance of the end target and the start target changing back to blue.

### Experiment 1: Bayesian sensorimotor learning (BSL)

**Figure 2.**
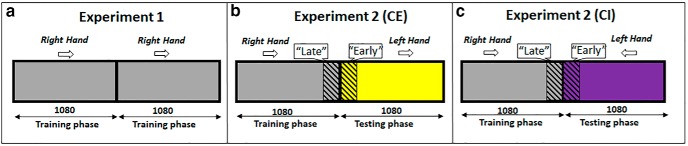
Experimental design. ***A***, In experiment 1, training and testing phases were nominally defined as the first and last 1080 trials, respectively. ***B***, ***C***, In experiment 2, the training phase consisted of 1080 RH trials followed by a testing phase of 1080 LH trials. In the congruent-extrinsic (CE) condition, the imposed visuomotor perturbation was a rightward lateral shift for both RH and LH trials. In the congruent-intrinsic (CI) condition the imposed visuomotor perturbation was a rightward lateral shift for RH trials and a leftward lateral shift for LH trials, both of which require elbow flexion. Mean endpoint ($\sigma_{\infty }$) for trials 980–1080 (RH late training) was used to compute the percentage of adaptation. The percentage of IG was computed by dividing the mean endpoint ($\sigma_{\infty }$) for trials 980–1080 (RH late training) by the mean endpoint ($\sigma_{\infty }$) for trials 1080–1180 (LH early testing).

The primary aim of experiment 1 was to test whether subjects learn to compensate for the imposed stochastic visuomotor perturbation (lateral shifts drawn from a distribution with fixed mean and SD) so that we could then probe whether, and the conditions under which, this learning generalized to the untrained limb. A secondary aim was to provide a close or approximate replication of the findings reported by Körding and Wolpert (2004). Before the experiment started, each subject performed 10 familiarization trials in which cursor feedback was always provided and no lateral shift was imposed. Further, for two of the seven BSL subjects tested, an additional Baseline task was run to measure each subject’s baseline motor variability and directional biases when reaching with each hand. The Baseline task used the same basic paradigm as the other experiments and consisted of the following sequence: 10 right hand (RH) feedback trials (cursor feedback always provided; no lateral shift imposed), 10 RH no-feedback trials (no cursor feedback provided; no lateral shift imposed), 10 left hand (LH) feedback trials (cursor feedback always provided; no lateral shift imposed), and 10 LH no-feedback trials (no cursor feedback provided; no lateral shift imposed). After completing the Familiarization and Baseline tasks, all subjects completed 2160 trials of the task with their RH (Fig. 2*A*,*B*). To preserve the 3:1:1:1 ratio between visual feedback conditions, we ran 1080 trial blocks (540 $\sigma_{0}$:180 $\sigma_{M}$:180 $\sigma_{L}$:180 $\sigma_{\infty }$). For the purposes of comparison with Körding and Wolpert (2004), and comparison with data from the IG experiment (experiment 2) we nominally defined the training phase as the first 1080 trials in each session and the testing phase as the second 1080 trials in each session (Fig. 2*A*). There were no objective differences between these phases in the experiment.

### Experiment 2: IG

**Figure 3.**
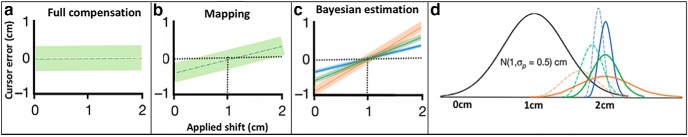
Computational models considered for experiment 1. The average lateral cursor deviation from the target (cursor error) as a function of the imposed shift for the models. Full compensation model (***A***), minimal mapping model (***B***), and Bayesian estimation model (***C***). (Transparent bands indicate the relative degree of variability in estimation.) The colors of the linear fits correspond to the visual condition (matching Fig. 1*H*), as do the bands of variability in ***C***. ***D***, The experimentally imposed prior distribution of shifts is Gaussian with a mean of 1 cm (in black). The probability distribution of possible visually experienced shifts under the clear, moderate, and large uncertainty conditions are represented with solid lines (colors as in Fig. 1*H*) for a trial in which the imposed shift is 2 cm. The Bayes-optimal estimate of the shift that combines the prior with the evidence is represented by dashed lines (colors also as in Fig. 1*H*). After Körding and Wolpert (2004).

The aim of experiment 2 was to build on the results of experiment 1, and test whether the learning exhibited in the BSL task generalizes from one limb to the other. Further, we were interested in testing whether the initial visuomotor learning that occurs during training is represented in extrinsic or intrinsic coordinates. Like experiment 1, experiment 2 started with 10 trials of a familiarization task in which cursor feedback was always provided and no lateral shift was imposed. After completing a training phase with their RH (1080 trials), subjects completed a testing phase (1080 trials) using their LH in which they experienced cursor feedback sampled from the same Gaussian distribution as experienced previously with the RH (mean of 1 cm, SD of 0.5 cm; Fig. 3*D*). To assess the reference frame in which transfer occurs, seven subjects experienced a congruent-extrinsic (CE) condition in which the cursor was shifted in the same visual direction across both the training phase with the right arm (Fig. 1*C*) and the testing phase with the left arm (Fig. 1*D*). By design, the imposed visuomotor perturbation was congruent in extrinsic (screen-based) coordinates (rightward lateral shift), yet incongruent in intrinsic coordinates (requiring an elbow joint flexion in the right arm and an elbow joint extension in the left to compensate for the shift). Another seven subjects experienced a congruent-intrinsic (CI) condition in which the cursor was shifted in opposite visual directions for each arm, and more specifically a rightward shift for the right arm during the training phase (Fig. 1*E*) and leftward shift for the left arm during the testing phase (Fig. 1*F*). This time, the visuomotor perturbation imposed across both the training and testing phases was congruent in intrinsic coordinates (requiring joint flexion in both right and left arms), yet incongruent in extrinsic coordinates.

### Data analysis

Kinematic data including hand position and velocity was recorded for all trials using BKIN’s Dexterit-E experimental control and data acquisition software (BKIN Technologies). Hand position data were recorded at 200 Hz and logged in Dexterit-E. Custom scripts for data processing and analysis were written in MATLAB. Hand position, velocity, and cursor shift values were extracted from the c3d files in MATLAB. A combined spatial- and velocity-based criterion was used to determine movement offset and corresponding reach endpoints (Georgopoulos et al., 1982; Moran and Schwartz, 1999; Scott et al., 2001). Specifically, movement offset was defined as the first point in time *t* at which the movement dropped below a minimum velocity threshold (<5% of peak velocity), after a minimum reach of 19 cm from the start target in the *y*-plane. Reach endpoints were defined as the *x* and *y* values at time *t*. The additional spatial criterion ensured that data from the start of the trial (also <5% of peak velocity) was not included in subsequent analysis.

Since the visual shift was systematically applied along the *x*-axis, the primary measure of the subject’s estimate of the visuomotor perturbation (the estimated prior) was their mean hand position (*x*-coordinate only) at the end of the reach (henceforth *endpoint*) for all reaches completed during the unlimited uncertainty ($\sigma_{\infty }$) condition. Because no midpoint feedback is provided during unlimited uncertainty ($\sigma_{\infty }$) trials, these provide a relatively uncontaminated measure of the estimated prior. In experiment 1, the mean endpoint was computed across the entire testing phase (trial 1080–2160; testing phase; Fig. 2*A*). In experiment 2, the degree of generalization was assessed by comparing the mean endpoint ($\sigma_{\infty }$) at the end of the training phase (trial 980–1080; late training phase) with the mean endpoint ($\sigma_{\infty }$) at the start of the testing phase (trial 1080–1180; early testing phase) for the respective groups.

The second measure of statistical learning was cursor deviation from target at movement offset as a function of the applied shift (cursor error). We compared the slopes of the linear fits for these plots, stratified by visual uncertainty condition, to determine the degree to which subjects compensated for visual uncertainty by changing their reliance on their stored prior (Körding and Wolpert, 2004). In experiment 1, cursor error as a function of shift (slope) was determined by averaging across the entire testing phase (trials 1080–2160). If subjects compensate fully for the visual feedback, then the average deviation from target for all conditions in which visual feedback is provided should be zero. If, however, subjects integrate both the learned prior and current visual evidence while performing the task, then endpoints should be biased toward the mean of the prior and should depend on sensory uncertainty (Körding and Wolpert, 2004). Accordingly, reach endpoints should be more biased toward the mean of the prior when sensory uncertainty is high (reflecting a higher weighting of this information) than when sensory uncertainty is low (reflecting a stronger reliance on or lower weighting of this information). Hence, if subjects perform Bayesian estimation, a linear relationship is predicted between cursor error and the imposed shift. More specifically, the linear fit should intercept the abscissa at the mean of the prior (1 cm) and have a slope that increases as a function of visual uncertainty.

A repeated measures ANOVA with planned pairwise comparisons was used to analyze mean endpoints across all subjects within experimental groups and the slopes for all experiments. The Mauchley test was used to assess the sphericity of repeated measures effects of visual condition as it constitutes a four-level factor. If sphericity was violated, Greenhouse–Geisser degree of freedom corrections were applied. The significance level for all non-corrected contrasts was α < .05. Statistical analysis was performed using SPSS v22.0 for Windows.

The same dependent measures were also used to test IG in experiment 2. The predictions of experiment 2 relate to the reference frame in which the initial learning occurred. If the learning that occurs during the training phase is represented in an extrinsic reference frame, this predicts that generalization will be relatively strong in the CE condition and relatively weak in the CI condition. If the learning that occurs during the training phase is represented in an intrinsic reference frame, this predicts that generalization will be relatively strong in the CI condition and relatively weak in the CE condition. IG was quantified according to the following generalization equation (Shadmehr and Mussa-Ivaldi, 1994; Wang and Sainburg, 2005; Brayanov et al., 2012):

(1) $$\%\text{Generalization=}\frac{\text{mean}\;\text{early}\;\text{LH}\;\text{endpoints}\;}{\text{mean}\;\text{late}\;\text{RH}\;\text{endpoints}\;}\times \text{100}$$

Finally, it is important to note that since we were primarily interested in assessing the degree of generalization of the learned prior, only endpoints from the ($\sigma_{\infty })$ trials were used in the analysis (as in experiment 1). In these trials, the influence on the prior is relatively uncontaminated by current sensory evidence (see above, Experimental procedures).

### Model predictions

For experiment 1, following Körding and Wolpert (2004), we considered three models of sensorimotor integration reflecting different computational strategies that subjects could use to reach accurately to the target on the basis of the visual feedback provided. One possibility is that subjects fully compensate for the sensed lateral shift (full compensation model; Fig. 3*A*). According to this model, increasing the uncertainty of the feedback for an imposed shift would increase endpoint variability (variance) without changing the mean. Importantly, this model does not require subjects to estimate either visual uncertainty or the prior distribution of shifts applied. The minimal mapping model involves an iterative mapping from visual feedback about cursor error to an estimate of the imposed shift. This crucial error signal can be reduced over repeated trials, and an accurate estimate of the shift can be attained. While this model predicts a mean endpoint of 1 cm to the left of the target (for a 1 cm rightward shift), indicating that the mean of the prior had been learned, it does not require an explicit representation of either the prior distribution or visual uncertainty (Körding and Wolpert, 2004). All that is required to learn this mapping is information about cursor error at the end of the movement. However, in our paradigm, cursor error is only provided for the clear feedback condition ($\sigma_{0}$). Therefore, a mapping may only be learned based on this condition and then applied to all other conditions ($\sigma_{M},\sigma_{L},\sigma_{\infty };$ hence the term minimal, for minimal condition mapping). Importantly, the minimal mapping model predicts a compensation pattern that is the same for all trials, regardless of visual uncertainty (Fig. 3*B*). The final model we considered is the Bayesian estimation model, according to which subjects use information about the prior distribution and the uncertainty associated with the visual feedback to estimate the imposed shift. The posterior probability distribution can be obtained by applying Bayes’ rule as follows:

(2) $$P\left(x_{true}|x_{sensed}\right)=\frac{P\left(x_{sensed}|x_{true}\right)P(x_{true})}{P\left(x_{sensed}\right)}$$

Where $x_{true}$ is the imposed shift, $x_{sensed}$ is the sensed shift (the visual evidence), and $P(x_{true})$ is the prior distribution of shifts. Assuming that the noise of each measurement is independent and Gaussian (Fig. 3*D*) then the optimal estimate of the imposed shift is a sum of the mean of the prior and the sensed feedback position$\left(\mu_{estimate}\right)$ weighted by their relative variances [($\sigma_{p}^{2})$and $\left(\sigma_{s}^{2}\right)$, respectively]:

(3) $$\mu_{estimate}=\frac{\sigma_{s}^{2}}{\sigma_{s}^{2}+\sigma_{p}^{2}}[1cm]+\frac{\sigma_{p}^{2}}{\sigma_{s}^{2}+\sigma_{p}^{2}}x_{sensed}$$

Where ($\frac{\sigma_{s}^{2}}{\sigma_{s}^{2}+\sigma_{p}^{2}}$) and $(\frac{\sigma_{p}^{2}}{\sigma_{s}^{2}+\sigma_{p}^{2}}$) is the “weighting” (degree of influence) attributed to the prior and visual information relative to their respective variance. Accordingly, the joint variance ($\sigma_{sp}^{2})$ of the posterior is given by:

(4) $$\sigma_{sp}^{2}=\frac{\sigma_{s}^{2}\sigma_{p}^{2}}{\sigma_{s}^{2}+\sigma_{p}^{2}}$$

The Bayesian estimation model predicts that as visual uncertainty increases, the subject’s estimate of the imposed shift moves away from the sensed shift and tends toward the mean of the learned prior distribution (Fig. 3*D*). For example, consider an imposed shift of 2 cm. Given sensory uncertainty there are multiple shifts that can produce a sensed shift of ∼2 cm (i.e., within the range of 1.8–2.2 cm). However, if visual uncertainty is a function of Gaussian noise on the visual feedback, then, according to the Bayesian model, the most probable shift is <2 cm, due to the influence of the learned prior. Hence, the estimated shift will tend toward the prior by an amount that depends on both the prior distribution and the degree of uncertainty in the visual feedback (Fig. 3*D*). Furthermore, without visual feedback ($\sigma_{\infty }$) the estimate should approximate the mean of the learned prior (because the likelihood distribution is flat).

**Figure 4.**
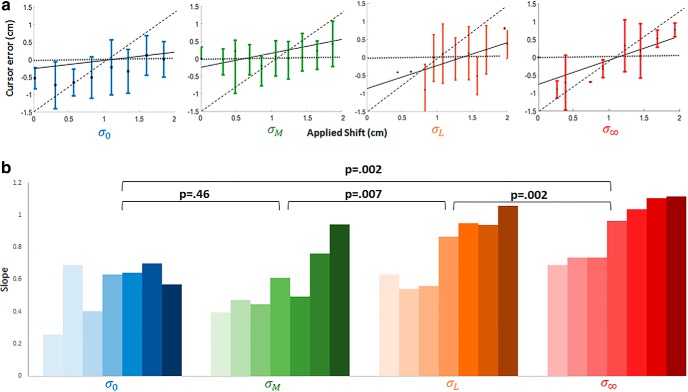
Effect of visual uncertainty. ***A***, Cursor error at the end of the trial as a function of the imposed shift for a representative subject. Colors as in Figure 1*H*. Values represent Cartesian (screen) coordinates. Horizontal dotted lines indicate the full compensation model prediction and diagonal dashed lines indicate the minimal mapping model prediction. Solid lines provide the Bayesian estimation model fits to the data as a function of sensory uncertainty. Due to trial scheduling statistics, applied shift values differ slightly across each subject. To reflect this difference, error bars denote SD instead of SEM. Importantly, every subject experienced the same overall statistical distribution of shifts during training and testing. ***B***, Slopes of the linear fits for all subjects in experiment 1. The first bar in each grouping corresponds to the subject represented in panel ***A***.

Based on the previous results of Körding and Wolpert (2004), we predicted that subjects would not only learn the prior distribution of imposed shifts but would apply it in a fashion consistent with the Bayesian estimation model. Accordingly, we predicted that the (sign-inverted) mean endpoint across the entire testing block (trials 1080–2160) would closely approximate the mean of the learned prior of 1 cm, and that subjects would integrate the degree of visual uncertainty when estimating the imposed shift. It was also expected that cursor error would increase as a function of increasing visual uncertainty as depicted in Figure 4*A*, where increasing error is indicated by a larger slope. That is, subjects will estimate the imposed shift with a greater degree of accuracy during trials in which visual feedback is more reliable, and with accuracy decreasing across less reliable visual feedback conditions (accuracy during σ_0_ > σ_*m*_ > σ_*L*_ > σ_∞_).

## Results

### Experiment 1

The mean endpoint ($\sigma_{\infty }$) across the experimental group was -1.51 ± 0.15 cm (mean ± SD) to the left of the target indicating that subjects had learned to compensate for the average experienced shift of +1 cm (mean of the imposed prior) over the ensemble of trials. As full compensation for the average shift would be -1 cm, the observed mean overshoot of -0.51 cm was unexpected. One possible explanation for this relates to the size of the cursor relative to that of the reach target and the associated spatial tolerance built into correct trials. The use of a 2 cm in diameter reach target and a 1 cm in diameter cursor meant that although subjects were instructed to reach as accurately to the target as possible, all trials in which the cursor stopped anywhere within the circumference of the target were counted as correct. Any pre-existing bias in any direction up to ± 1 cm (the radius of the target) might therefore remain uncorrected through the experiment. To determine whether this was the case, we collected baseline reach data for the last two of the seven subjects in experiment 1 and performed baseline corrections on their endpoint data. Baseline-adjusted mean endpoint averaged over those two subjects indicate a compensation of -1.16 ± 0.12 cm (mean ± SD) providing a closer correspondence to the mean of the true prior and the results reported by Körding and Wolpert (2004).

Next, we examined the relationship between the imposed shift and cursor error (Fig. 4). Cursor error as a function of shift was averaged across 11 bins of applied shift values and plotted for all visual feedback conditions (representative subject in Fig. 4*A*). The slope of the linear fit was analyzed to investigate the relationship between cursor error and the imposed shift (Fig. 4*B*). Mauchly’s test for sphericity was violated (*p* = .01), requiring Greenhouse–Giesser correction. According to the corrected repeated measures ANOVA, slope increased significantly (*F*_(3,81)_ = 14.1 *p* = .002) with increasing uncertainty in the visual feedback. A planned comparison of the slopes between visual conditions indicated significant differences for all visual conditions except the $\sigma_{0}$ conditions which was similar (*p* = .46) to condition $\sigma_{M}$. This pattern, in which reliance on the learned prior is inversely related to changes in visual uncertainty, is consistent with the Bayesian estimation model and inconsistent with both the full compensation and minimal mapping models.

The influence of visual uncertainty on cursor error was also evident when averaged across all the subjects tested (*n* = 7). The mean slope increases significantly with increasing visual uncertainty across three of the conditions (σ_*M*_, σ_*L*_, σ_*∞*_), although not for the clear condition (σ_0_); Fig. 4*B*). One possible explanation for this is that the clear and moderate uncertainty conditions provide highly similar information about the imposed shift (Fig. 1*H*). Although the stimuli used for the moderate uncertainty condition were randomly generated Gaussian point cloud distributions of 25 small translucent spheres with a SD of 1 cm, the origin of the moderate uncertainty feedback is, on qualitative inspection, still relatively easy to discern. Hence, from the subject’s perspective, there might have been little effective difference between the clear and moderate uncertainty feedback conditions, which could have produced the similar slopes observed across these two conditions. Nevertheless, the influence of visual uncertainty on cursor error remains significant for all other comparisons.

### Experiment 2

**Figure 5.**
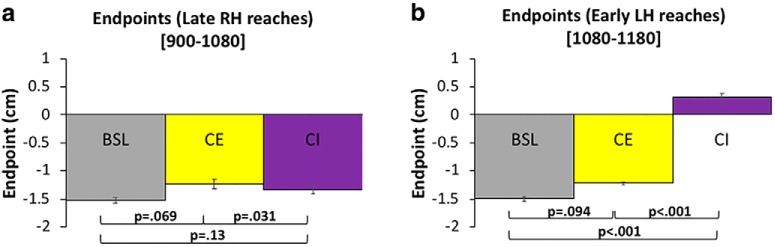
A comparison of endpoints ($\sigma_{\infty }$) for all experimental groups. ***A***, Mean endpoint during late RH training. ***B***, Mean endpoints during early LH testing. All *p* values represent significance levels of independent samples *t* tests. Error bars denote SEM. Color coding is the same as in Figure 2.

In this experiment, a training phase of 1080 RH trials was followed by 1080 LH trials for both the CE and CI conditions (Fig. 2*B*,*C*). The percentage of IG was determined by comparing the mean endpoint ($\sigma_{\infty }$) during late training (trials 980–1080) against the mean endpoint ($\sigma_{\infty }$) from the early LH trials (1080–1180), as per Equation 1. To rule out the possibility that training differences between subjects participating in Experiments 1 and 2 could influence our results, mean endpoints during late RH training (980–1080) were compared across all experimental groups. Endpoints were similar: -1.52 ± 0.2, -1.23 ± 0.32, and -1.34 ± 0.22 cm (mean ± SD in all cases) for all groups (Fig. 5*A*), and the observed differences were not significant (BSL vs CE, *p* = .069; CE vs CI, *p* = .31; BSL vs CI, *p* = .15). This result indicates that learning of the prior distribution for CE and CI subjects in experiment 2 was comparable to the learning that occurred for subjects in experiment 1. In addition, the mean endpoint during late training (980–1080) for BSL subjects was similar to both late training (1980–2160), and the entire block of 1080 trials from the testing phase (early vs late, *p* = .63; early vs entire, *p* = .99; late vs entire, *p* = .64), thus reinforcing the use of late testing reaches (980–1080) as a suitable indicator of prior learning.

During early LH trials (1080–1180), a mean endpoint of -1.22 ± 0.1 cm (mean ± SD) was observed for subjects in the CE group, which is similar to BSL endpoints during the same period (*p* = .094; Fig. 5*B*). This indicates strong (98%) generalization of the learned prior when the visual perturbation is congruent in an extrinsic reference frame. In contrast, an endpoint of 0.31 ± 0.26 cm (mean ± SD) during early LH reaches was observed for CI subjects, which is significantly different to both CE and BSL endpoints during the same period (*p* = .0001 in both cases; Fig. 5*B*). This indicates that the learned prior incompletely generalized (23%) when the perturbation is congruent only in an intrinsic reference frame.

**Figure 6.**
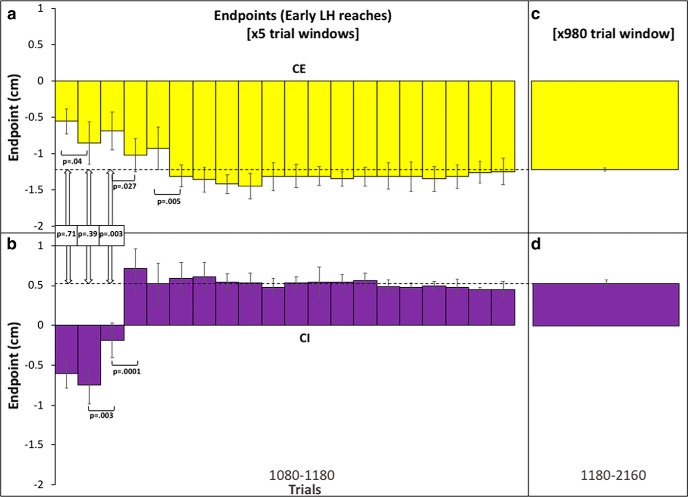
Moving average plot for early LH ($\sigma_{\infty }$) trials. ***A***, ***B***, A moving average of endpoints across the first 100 trials in the LH testing phase for CE (***A***) and CI (***B***). Each bar represents the average across seven subjects using a window size of five trials. Error bars denote SD not SEM to reflect the disparate number of values included in each window. All *p* values represent significance levels from independent Welch *t* tests. ***C***, ***D***, Average endpoints for CE (***C***) and CI (***D***) conditions for the remaining reaches in the testing phase (1180–2160). In ***C***, ***D***, error bars denote SEM. The dashed line represents the mean across reaches 1180–2160. Color coding is the same as in Figure 2.

Although the mean endpoint averaged across the first 100 trials of the testing phase provides one measure of generalization, it may also reflect some degree of new learning with the opposite limb. We therefore performed a moving average window analysis (window size = five trials) on early LH reaches (1080–1180), which provides a higher temporal resolution measure (Fig. 6*A*,*B*). We also ran a *post hoc* analysis which confirmed that the selected window size provided a representative (relative frequency-preserving) sampling of visual uncertainty conditions including $\sigma_{\infty }$ trials and representative distributions of shift values. For the CE group, the moving average shows a mean endpoint of -0.56 ± 0.17 cm (45%) over the first five trials, -0.86 ± 0.29 cm (68%) over the next five trials, before the mean endpoints stabilize from trial 30 onwards and become statistically indistinguishable across the remaining trial windows in the testing phase (1180–2160). The mean endpoint during this part of the testing phase was -1.22 cm ± 0.24 cm (Fig. 6*C*). For the CI group, the moving average shows a mean endpoint of -0.60 ± 0.18 cm (45%) in the first five trials and -0.75 ± 0.23 cm (55%) between 5 and 10 trials, with endpoints reaching a plateau at a mean of 0.37 ± 0.14 cm (30%) after 100 trials (Fig. 6*B*) which persisted for the remainder of the testing phase. The mean endpoint during this part of the testing phase was 0.51 ± 0.19 cm (Fig. 6*D*).

Interestingly, there was no significant difference between mean endpoints for both CE and CI groups after five trials (*p* = .71) or after 10 trials (*p* = .39). Both CE and CI groups show the same percentage generalization of the prior mean (45%) during early LH reaches. Importantly, this pattern of results is not readily interpretable in terms of new learning or adaptation with the opposite limb. Since new learning would be expected to occur in the same reference frame as initial learning, this should produce endpoints that diverge rather than converge across the CE and CI groups. For example, if initial and new learning of the imposed perturbation are encoded in extrinsic coordinates, endpoints should tend toward -1 cm for the CE group and +1 cm for the CI group. By contrast, if initial and new learning are encoded in intrinsic coordinates, endpoints should tend toward +1 cm for the CE group and -1 cm for the CI group. No divergence in endpoints was observed during early LH reaches. Instead, a significant difference between CE and CI groups only begins to emerge after 15 trials (*p* = .003) and is maintained over the remaining trials, indicating that new learning involving the opposite limb does eventually take place.

## Discussion

In the current study, we demonstrate that subjects integrate their current level of uncertainty about the available visual evidence with the prior distribution learned from the task to generate motor behavior that optimally compensates for the imposed shift. In other words, they perform Bayesian integration during sensorimotor learning. Our results therefore replicate those of Körding and Wolpert (2004). We also wanted to go beyond simply replicating these important results by further probing the nature of the underlying representations that are learned during the task. By investigating generalization of the learned prior distribution to the other limb, we were able to assess the reference frame in which initial learning occurs.

Both early CE and CI endpoints suggest the prior learned during RH adaptation is encoded in an extrinsic reference frame and this representation is available to the opposite limb. This finding parallels a number of earlier results demonstrating that intralimb generalization of visuomotor perturbations occurs in extrinsic coordinates (Flanagan and Rao, 1995; Wolpert et al., 1995; Vetter et al., 1999; Krakauer et al., 2000) and IG occurs in extrinsic coordinates (Imamizu and Shimojo, 1995; Sainburg and Wang, 2002; Wang and Sainburg, 2004, 2005; Taylor et al., 2011). Nevertheless, it differs from several more recent findings. For instance, Carroll et al. (2014) report strong and immediate interlimb transfer only when the (non-stochastic) visuomotor perturbation was congruent across both intrinsic and extrinsic reference frames. Transfer was limited when the visuomotor perturbation was only congruent in a single reference frame (19% during an extrinsic-congruent condition and 8% during an intrinsic-congruent condition). An important difference between the paradigm employed by Carroll et al. (2014) and the current study is that they used an isometric force aiming task in which subjects generated small forces or torques in their index finger that were in turn mapped into cursor movements. Isometric movements, which by definition involve muscle contraction without corresponding changes in joint angle and muscle length, differ from natural multi-joint movements both in terms of muscle activity and proprioception (Sergio et al., 2005). It is therefore plausible that visuomotor learning in an isometric task, which involves learning new dynamics, more likely requires coding in intrinsic, joint-based coordinates (Shadmehr and Mussa-Ivaldi, 1994; Rotella et al., 2015). Because our paradigm does not require subjects to learn a new mapping from forces into cursor movements, this could explain the lack of alignment between our findings and those of Carroll et al. (2014).

More recently, Poh et al. (2016) report stronger transfer when the visuomotor perturbation is congruent in both extrinsic and intrinsic coordinates as compared to when the perturbation is aligned in only a single extrinsic or intrinsic reference frame. Because they used a more standard (non-isometric) reaching task, the above considerations do not apply. Although their results appear at odds with those reported here, one important difference is that they focus on the degree of transfer relatively late in the testing phase with the untrained limb (specifically the last two blocks of “probe” trials). By contrast, our primary focus was on early transfer. Although paradigm differences make a direct comparison difficult, the pattern of transfer Poh et al. (2016) observed in early blocks of probe trials appears less consistent with their mixed reference frame conclusion than the transfer pattern observed in late blocks (Poh et al., 2016, their Fig. 4, p 1245).

Several other features of our IG data warrant discussion. One especially striking feature is the rapid adaptation following immediate generalization observed in both CE and CI groups. This may suggest the operation of cognitive strategies or heuristics, which can occur at fast timescales (Malfait and Ostry, 2004; Hwang et al., 2006). It has recently been argued that generalization between effectors and across workspaces may involve both implicit and explicit learning processes (Taylor and Ivry, 2013; Taylor et al., 2014; McDougle et al., 2015). With these distinct learning processes in mind, Poh et al. (2016) investigated the contribution of explicit processes to the transfer of visuomotor learning and found that explicit learning is typically encoded in extrinsic coordinates and is fully available early during opposite limb reaches. If explicit cognitive strategies are recruited, an early and abrupt error-corrective switch is predicted corresponding to the time at which subjects explicitly recognize a change in task context and adopt a novel explicit strategy (e.g., reach to the right of the target). This explicit strategy might help subjects achieve relatively rapid compensation in the task in contrast to the slower learning expected if an implicit, error-based process is exclusively relied on. Although an interesting source of speculation, the current paradigm was not designed to disentangle implicit and explicit learning processes.

Another interesting result is the learning plateau exhibited in the CI reaches over the course of the testing block (Fig. 6*D*). One possible explanation for this CI-specific effect might be anterograde interference. Interference has been demonstrated when a counter-rotation equal in magnitude but opposite in direction is learned shortly after an initial visuomotor rotation is learned and consolidated in memory (Wigmore et al., 2002; Krakauer et al., 2005). Once consolidation occurs, the newly acquired internal model is thought to become increasingly resistant to modification by a competing model (Krakauer, 2009). Not only does consolidation commence rapidly (significant consolidation after ∼5 min), but it appears to strengthen as a function of time and is strongly correlated with number of adaptation trials performed (Krakauer et al., 2005). Given that our subjects adapted to the extrinsically encoded perturbation over a large number of trials (n = 1080), consolidation may make the learned prior more resistant to change. Accordingly, consolidation predicts rapid and early stabilization toward a mean endpoint of -1 cm over the course of LH reaches for the CE group. In contrast, for the CI group, it is plausible that the consolidated prior is erroneously applied to visual shifts that are incongruent in extrinsic coordinates and remains difficult to unlearn leading to the observed plateau at a mean of 0.51 cm by the end of the testing block. Additional experiments are required to determine whether the anterograde interference hypothesis has merits.

Although the current study provides valuable information about the reference frame in which the mean of the learned prior generalizes across limbs, a number of important questions remain open. Since we were interested in replicating the results of Körding and Wolpert (2004), elements of their paradigm were preserved in our extension to the context of IG which placed limitations on the questions we could probe. For example, the current paradigm did not allow us to ask whether immediate generalization increases when the imposed visuomotor perturbation is congruent across both extrinsic and intrinsic reference frames (Carroll et al., 2014). Another limitation is that our study, like that of Körding and Wolpert (2004), was not designed to address the extent to which the visual likelihood was learned (Sato and Körding, 2014). Relatedly, our paradigm was not optimised to investigate likelihood integration when subjects switched to the untrained limb. Since generating our slope plots (Fig. 4) requires a full Gaussian distribution of imposed shift values for each visual uncertainty condition, it was not possible to address this question with the current design. Future studies with modified designs are required to address these and other important questions about Bayesian integration in sensorimotor learning.

In this study, we extended the findings of Körding and Wolpert (2004) to the context of interlimb generalization. We found that, in our task, the learned prior is available to the untrained limb and is coded in an extrinsic reference frame. These findings open pathways for future investigation into the nature of statistical learning in sensorimotor adaptation.

## Synthesis

Reviewing Editor: Li Li, New York University Shanghai Decisions are customarily a result of the Reviewing Editor and the peer reviewers coming together and discussing their recommendations until a consensus is reached. When revisions are invited, a fact-based synthesis statement explaining their decision and outlining what is needed to prepare a revision will be listed below. The following reviewer(s) agreed to reveal their identity: Iris Vilares.

Both reviewers believe the ms addressed an important question in the field. However, they both raised concerns that should be addressed before the ms can be considered for publication.

Reviewer 1:

In the present study, the authors studied inter-manual transfer of learned visuomotor adaptation under the Bayesian integration framework first proposed by Körding and Wolpert (2004). First, the authors largely replicated original results of Körding and Wolpert 2004, using a different experimental apparatus. Then, they further found that the learned adaptation skill can transfer from right arm to left arm with comparable accuracy, and that such comparable accuracy only occurred for the extrinsic coordinates (same adaptation direction) but not for the intrinsic coordinates (different adaptation direction).

Overall, this study made a novel contribution to visuomotor adaptation using Bayesian framework, and experimental design and data analysis are clear and convincing.

General comments:

1. The authors mentioned the KINARM robot was utilized in the footnote. More rationale may be needed as of why such device can achieve similar result as the optical tracking system used in Körding and Wolpert 2004.

2. This work is based on a large number of experimental trials: 1080 for all training stages. Therefore, besides the perturbation prior, other aspects of the motor system may have also been learned and consolidated in the training process, such as sensory likelihood. It would be better to clarify in Discussion.

3. The authors discussed the use of Late and Early stages in Results of Experiment 2, but logically this may also be stated earlier in the experimental design section. These stages are important for the “early transfer” which is an important setting of this work, and therefore the authors may clearly and explicitly explain the purpose of designing such special stages.

Specific comments:

1. Figure caption is missing for Figure 2.

2. “bayesian”in the title should be capitalized in the initial letter.

3. Page 6, main text, line 3, x cm

4. Page 8, main text, line -6, 3:1`:1:1

5. Page 18, line 1-2, missing reference for Bayesian Estimation Model and Full Compensation and Minimal Mapping Model (Körding and Wolpert 2004?).

Reviewer 2:

In this paper the authors set out to: 1) Replicate the key findings of Kording & Wolpert (2004) in terms of how people perform in a target-shift visuomotor task (if Bayesian or not); and 2) Understand if learning in a visuomotor task generalizes from one limb to the other and, if yes, if it does so in intrinsic and/or extrinsic coordinates.

They were able to replicate the key findings of Kording & Wolpert, and also found that visuomotor learning in one limb can generalize to the other limb. Furthermore, the consistent generalization just happened when the perturbations imposed in the task followed an extrinsic reference frame, suggesting that learning in the task occurred in extrinsic coordinates.

The questions in the paper are pertinent and well defined, and the methods appropriate to address them. The paper is well-written and generally clear. I particularly enjoyed the methods section and the (generally) clear explanations provided therein. I have, though, a few suggestions/concerns that I believe could be addressed before the paper gets published:

Major:

- My main concern is with the number of participants per experiment (which for some of the experiments was only 7). While these participant numbers are common in the sensory-motor field, and hence the authors should not be penalized by following the field's convention, this limitation should be addressed in the discussion. Furthermore, as it is unlikely that 7 participants will be representative of the whole of human population, I would suggest rephrasing/toning down some of the extrapolations written in the text (e.g. in the discussion, I would suggest replacing “We found that the learned prior is available to the untrained limb and is coded in an extrinsic reference frame” with “We found that, in our task, the learned prior was available to the untrained limb and was coded in an extrinsic reference frame”...).

- How were participants recruited? Could this have created some bias?

- Results: In the results of Experiment 2, it says that the mean endpoints during the late RH training were “highly similar”. However, the mean endpoint of the first group (-1.52) was not that similar to the other two groups, and indeed almost significantly different (p = 0.069). While we cannot say that they were significantly different (which given the small sample size is not surprising), it is a bit misleading to state that they are “highly similar”. The same comment applies to the reporting of the comparison of mean endpoints during early LH trials. This could be further discussed;

- There were almost no limitations reported in the discussion section.

Minor:

- Methods: What is the color of the end target (the one participants have to reach)? Is it also red/blue? And what is the color of the cloud of feedback dots? The text refers translucent dots but it is unclear what the base color is (maybe white, if we go by Fig. 1h ?).

- LH/RH. I assume these stand for left hand (LH)/right hand (RH), but the first time they are used they should be spelt out, with the abbreviation in parenthesis.

- Figure 4: legend not very clear. Namely, in Fig. 4a, what do the error bars stand for? Fig 4b: it refers error bars but there are none. Are the error bars referred here the ones displayed in Fig. 4a? If yes then the explanation should go in the legend of 4a, not 4b, as it can be confusing otherwise. Fig 4b should have error bars nevertheless (associated with the slopes).

- Fig. 5 and Fig. 6: could benefit from additional editing to be clearer/more visually appealing (i.e. to be more like Figure 3 and 4).

- When was the additional Baseline Task done in 2 of the subjects performed, and did these participants also performed the test phase with the LH?
